# The association between systemic immune-inflammation index and chronic obstructive pulmonary disease in adults aged 40 years and above in the United States: a cross-sectional study based on the NHANES 2013–2020

**DOI:** 10.3389/fmed.2023.1270368

**Published:** 2023-11-24

**Authors:** Yifeng Xu, Zhaoqi Yan, Keke Li, Liangji Liu

**Affiliations:** ^1^School of Clinical Medicine, Jiangxi University of Chinese Medicine, Nanchang, Jiangxi, China; ^2^Department of Respiratory and Critical Care Medicine, Affiliated Hospital of Jiangxi University of Chinese Medicine, Nanchang, Jiangxi, China

**Keywords:** systemic immune-inflammation index, chronic obstructive pulmonary disease, NHANES, cross-sectional study, restricted cubic spline

## Abstract

**Background:**

Inflammation is the core of Chronic obstructive pulmonary disease (COPD) development. The systemic immune-inflammation index (SII) is a new biomarker of inflammation. However, it is currently unclear what impact SII has on COPD. This study aims to explore the relationship between SII and COPD.

**Methods:**

This study analyzed patients with COPD aged ≥40 years from the National Health and Nutrition Examination Survey (NHANES) in the United States from 2013 to 2020. Restricted Cubic Spline (RCS) models were employed to investigate the association between Systemic immune-inflammation index (SII) and other inflammatory markers with COPD, including Neutrophil-to-Lymphocyte Ratio (NLR) and Platelet-to-Lymphocyte Ratio (PLR). Additionally, a multivariable weighted logistic regression model was utilized to assess the relationship between SII, NLR and PLR with COPD. To assess the predictive values of SII, NLR, and PLR for COPD prevalence, receiver operating characteristic (ROC) curve analysis was conducted. The area under the ROC curve (AUC) was used to represent their predictive values.

**Results:**

A total of 10,364 participants were included in the cross-sectional analysis, of whom 863 were diagnosed with COPD. RCS models observed non-linear relationships between SII, NLR, and PLR levels with COPD risk. As covariates were systematically adjusted, it was found that only SII, whether treated as a continuous variable or a categorical variable, consistently remained positively associated with COPD risk. Additionally, SII (AUC = 0.589) slightly outperformed NLR (AUC = 0.581) and PLR (AUC = 0.539) in predicting COPD prevalence. Subgroup analyses revealed that the association between SII and COPD risk was stable, with no evidence of interaction.

**Conclusion:**

SII, as a novel inflammatory biomarker, can be utilized to predict the risk of COPD among adults aged 40 and above in the United States, and it demonstrates superiority compared to NLR and PLR. Furthermore, a non-linear association exists between SII and the increased risk of COPD.

## Introduction

Chronic obstructive pulmonary disease (COPD) is a heterogeneous lung disease characterized by persistent and often progressive airflow obstruction due to abnormalities of the airways and/or alveoli, leading to chronic respiratory symptoms such as dyspnea, cough, and expectoration (1). Currently, COPD has become the third leading cause of death worldwide, causing a huge economic burden. In the United States, COPD-related costs are expected to increase to $800.9 billion over the next 20 years, or $40 billion per year (2). At the same time, the heterogeneity and complex pathophysiological mechanisms of COPD pose great challenges to diagnosis and prognosis (3). The Global Initiative for Chronic Obstructive Lung Disease (GOLD) mentioned a “treatable trait” (TTs) in 2023, which may address this issue by identifying COPD patients with an increased risk of exacerbation through biomarker identification (4). The discovery of a reliable, measurable, and clinically relevant biomarker would be invaluable.

The systemic immune-inflammation index (SII), initially used as a prognostic indicator of adverse outcomes in cancer patients, is a new and stable systemic inflammation evaluation index calculated based on platelet count × neutrophil count/lymphocyte count (5). In recent years, the application of SII has been expanding to assess the severity of and monitor treatment effects for diseases such as coronary artery disease, rheumatoid arthritis, and liver fibrosis (6–8). Previous studies have shown that inflammation is the core of COPD development, and the infiltration of inflammatory cells can lead to the destruction of structural cells such as airway epithelial cells, matrix cells, and parenchymal cells, with the degree of airway inflammation increasing with the severity of COPD (9). Neutrophils are key inflammatory cells in the pathogenesis of COPD, and an increased number of neutrophils in the blood is a characteristic of all COPD patients. Some studies have suggested that elevated levels of the inflammatory markers neutrophil-to-lymphocyte ratio (NLR) and Platelet-to-Lymphocyte Ratio (PLR) may be associated with an increased risk of deterioration in COPD patients (10, 11). However, there is no research on the relationship between SII and COPD.

To address this knowledge gap, we carried out a cross-sectional study utilizing a substantial sample of individuals aged 40 years and older from the National Health and Nutrition Examination Survey (NHANES) to assess the association between SII levels and COPD. We also employed Receiver Operating Characteristic (ROC) curves to evaluate the predictive abilities of SII, NLR, and PLR for COPD risk in different models.

## Methods

### Study population

The NHANES study is a multistage, stratified, and nationally representative study of the US population. It is conducted by the National Center for Health Statistics of the Centers for Disease Control and Prevention, aimed at assessing the nutrition and health status of adults and children in the United States. The survey includes demographic data, dietary data, examination data, laboratory data, questionnaire data, etc. In this study, we selected publicly available data from NHANES 2013–2020. All procedures during the NHANES 2013–2020 cycle have been approved by the National Center for Health Statistics Research Ethics Review Committee and have obtained written informed consent from all participants.

**Figure 1:**
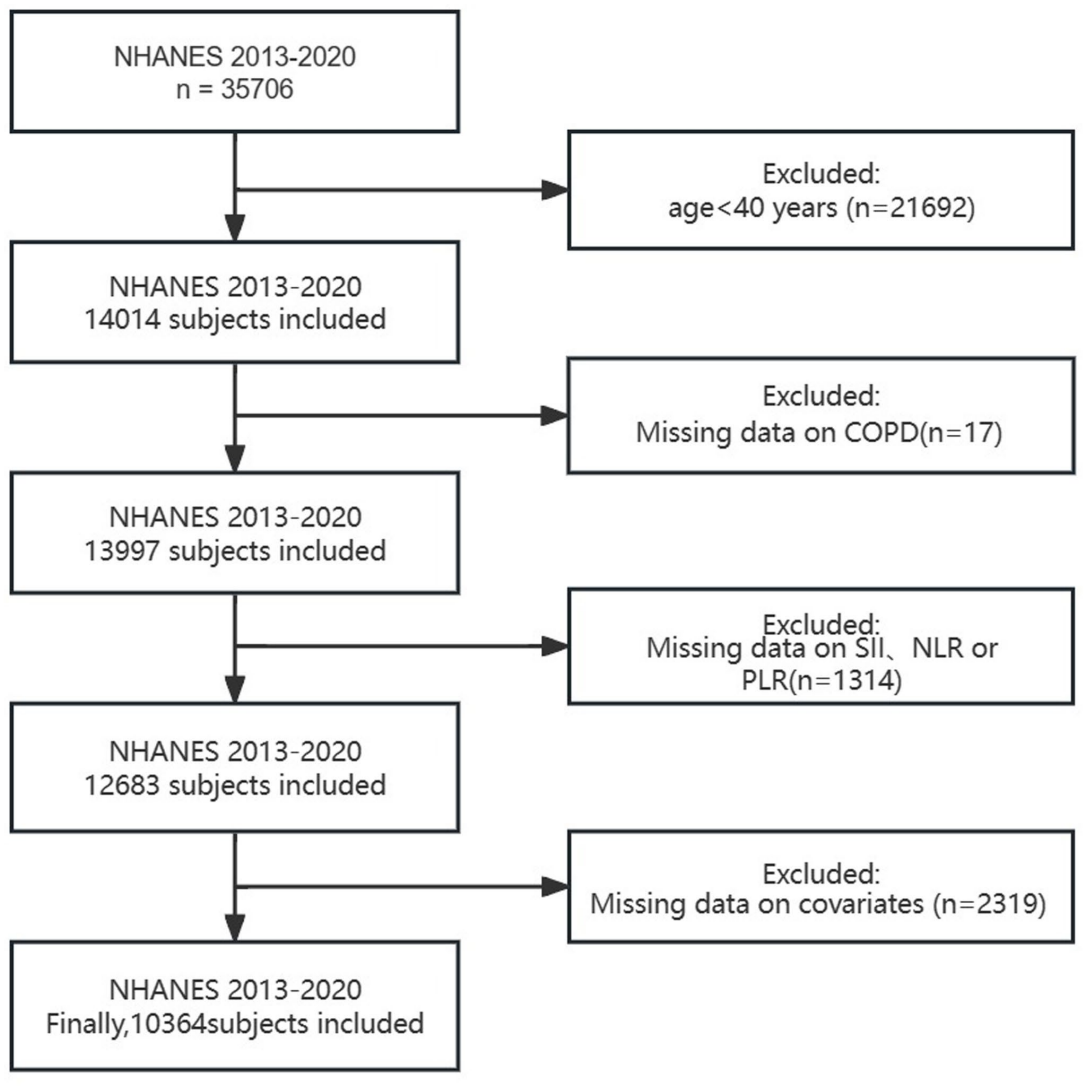
Flowchart of participant selection.

Our study excluded the following (Figure 1): (1) individuals under 40 years old; (2) individuals with missing COPD data; (3) individuals with missing SII、NLR or PLR data; (4) individuals with missing covariate data (such as smoking, BMI, hypertension, diabetes, etc.). Finally, a total of 10,364 people were included in this study. Considering that our study includes hematological variables, we chose the weights from the Mobile Examination Center (MEC).

### Assessment of COPD

The definition of COPD is a positive answer to any of the following questions: (1) “Has a doctor or other health professional ever told you that you have emphysema?” (2) “Has a doctor or other health professional ever told you that you have COPD?”

### Assessment of SII, NLR, and PLR

The SII, NLR, and PLR were used as the dependent variable in this study. The CoulterDxH 800 analyzer, an automated hematology analyzer, was used to measure lymphocyte, neutrophil, and platelet counts in whole blood, reported as 10^3^ cells/μL. The SII was calculated using the following formula: platelet count × neutrophil count/lymphocyte count (5). Additionally, we also considered NLR: neutrophil count/lymphocyte count and PLR: platelet count/lymphocyte count.

### Covariates definition

Our covariates include age, sex, race/ethnicity (Mexican American, non-Hispanic white, non-Hispanic black, non-Hispanic Asian, other Hispanic, Other/multiracial), education level (less than 9th grade, 9–11 grade, high school graduate, some college or AA degree, and college graduate or above), BMI, poverty-income ratio (PIR), serum cotinine (COT), smoking status, cardiovascular disease (CVD), hypertension, and diabetes. PIR was categorized into three groups: Low (≤1.39), Medium (>1.39, ≤3.49), and High (>3.49). Smoking status was classified as current, former, or never smoking; current smokers were defined as smoking more than 100 cigarettes but sometimes or continuously smoking, former smokers as smoking more than 100 cigarettes but not currently smoking, and never smokers as smoking less than 100 cigarettes in their lifetime. For CVD, a positive response to whether a doctor or other health professional has ever told you that you have congestive heart failure (CHF)/coronary heart disease (CHD)/angina/heart attack/stroke was defined as having CVD. Hypertension was defined based on the American Heart Association/American College of Cardiology (AHA/ACC) 2017 guidelines (12), as having a systolic blood pressure ≥ 130 mmHg or diastolic blood pressure ≥ 80 mmHg, with self-reported doctor diagnosis or with antihypertensive medications administrations. Diabetes was defined as self-reported diagnosis of diabetes and use of diabetes medication.

### Statistical analyses

Considering that the inflammatory markers SII, NLR, and PLR exhibited a right-skewed distribution, they were log2-transformed for regression analysis. The association between SII, NLR, and PLR with COPD was examined using a restricted cubic spline (RCS) regression model, with data fitted by a logistic regression model. This model was constructed with four knots at the 5th, 35th, 65th, and 95th percentiles of Log2-SII, Log2-NLR, and Log2-PLR (reference is the 5th percentile). Based on the results of the RCS analysis, the quartiles of Log2-SII, Log2-NLR, and Log2-PLR were assessed in relation to COPD, with the second quartile serving as the reference group.

Continuous variables are reported as mean ± standard deviation (SD), and categorical variables are presented as percentages. Weighted t-tests (for continuous variables) and weighted chi-square tests (for categorical variables) were used to assess differences between COPD and non-COPD subjects, while weighted Kruskal-Wallis tests (continuous variables) and weighted chi-square tests (categorical variables) were separately conducted to assess variations among the quartile groups of Log2-SII, Log2-NLR, and Log2-PLR. The weighted t-test takes into account the variance differences between samples, providing a more precise comparison of the mean differences between two groups. Additionally, for categorical variables, the weighted chi-squared test can be employed for evaluation. The weighted chi-squared test considers differences in sample weights, thereby enabling a more accurate assessment of proportion differences between two groups. The Kruskal-Wallis test is a non-parametric method suitable for comparing median differences among multiple groups.

The association between Log2-SII, Log2-NLR, and Log2-PLR with COPD was further analyzed using multiple logistic regression analysis. Initially, a crude model was fitted without adjustment, followed by stepwise adjustment for covariates. Model 1 was adjusted for age, sex, and race; Model 2 included further adjustments for PIR, BMI, serum cotinine, education level, and smoking status in addition to Model 1; Model 3 included adjustments for cardiovascular disease, hypertension, and diabetes in addition to Model 2. The results were presented in terms of odds ratios (ORs) and their respective 95% confidence intervals (CIs). We used ROC curves to calculate the Area Under the Curve (AUC) to assess the predictive ability of SII, NLR, and PLR for COPD in different models.

Lastly, stratified analyses were carried out based on sex, age, BMI, cardiovascular disease, hypertension, and diabetes. We assessed the presence of interaction effects among these variables using interaction terms. The statistical analysis was conducted using R Studio (version 4.2.2), with a significance threshold set at *p* < 0.05 (two-sided).

## Results

### Basic characteristics of participants

A total of 10,364 COPD subjects (mean age 58.1 years, 52% male) were included, of which 863 (7.3%) were diagnosed with COPD. Compared with the non-COPD group, COPD subjects were more likely to be older, non-Hispanic white, have higher education levels, be more likely to smoke, and be more likely to be overweight/obese (BMI ≥ 25 kg/m^2^) and have hypertension. There were also statistically significant differences between the two groups in terms of PIR, serum cotinine, cardiovascular disease, and diabetes (all *p* < 0.05) (Table 1).

**Table 1:** Baseline characteristics of study participants by incident COPD.

Characteristic	Overall, *N* = 10,364 (100%)^1^	COPD, *N* = 863 (7.3%)^1^	Non-COPD, *N* = 9,501 (93%)^1^	*P-*value^2^
Age (years)	[58.1 (11.5)]	[63.8 (10.4)]	[57.7 (11.5)]	**<0.001**
Serum cotinine (ng/mL)	[56.5 (134.3)]	[132.9 (167.2)]	[50.6 (129.5)]	**<0.001**
BMI				**0.010**
Normal (<25)	2,455 (23.8%)	203 (25.0%)	2,252 (23.7%)	
Overweight (≥25, <30)	3,510 (34.3%)	237 (27.9%)	3,273 (34.8%)	
Obese (≥30)	4,399 (41.9%)	423 (47.1%)	3,976 (41.5%)	
Sex				0.4
Female	5,291 (52%)	427 (54%)	4,864 (52%)	
Male	5,073 (48%)	436 (46%)	4,637 (48%)	
Race				**<0.001**
Non-Hispanic White	4,231 (71%)	536 (81%)	3,695 (71%)	
Non-Hispanic Black	2,306 (9.7%)	158 (6.9%)	2,148 (9.9%)	
Mexican American	1,287 (6.3%)	36 (1.8%)	1,251 (6.7%)	
Non-Hispanic Asian	1,123 (4.8%)	21 (1.1%)	1,102 (5.1%)	
Other Hispanic	1,080 (5.0%)	57 (2.9%)	1,023 (5.2%)	
Other/multiracial	337 (3.0%)	55 (6.3%)	282 (2.7%)	
PIR				**<0.001**
High (>3.49)	3,487 (48%)	148 (24%)	3,339 (50%)	
Medium (>1.39, ≤3.49)	3,694 (33%)	315 (38%)	3,379 (32%)	
Low (≤1.39)	3,183 (19%)	400 (38%)	2,783 (18%)	
Education attainment				**<0.001**
Less than 9th grade	992 (4.8%)	70 (5.2%)	922 (4.8%)	
9–11th grade	1,206 (8.4%)	151 (15%)	1,055 (7.9%)	
High school grad/GED	2,396 (23%)	255 (34%)	2,141 (22%)	
Some college or AA degree	3,153 (31%)	280 (30%)	2,873 (31%)	
College graduate or above	2,617 (32%)	107 (15%)	2,510 (34%)	
Smoking status				**<0.001**
Current smoker	1,821 (16%)	322 (40%)	1,499 (15%)	
Former smoker	2,992 (30%)	353 (40%)	2,639 (30%)	
Never smoker	5,551 (53%)	188 (19%)	5,363 (56%)	
Cardiovascular disease	1,644 (13%)	352 (40%)	1,292 (11%)	**<0.001**
Hypertension	7,149 (64%)	678 (76%)	6,471 (63%)	**<0.001**
Diabetes	2,217 (17%)	278 (29%)	1,939 (16%)	**<0.001**
Log2-SII	[8.86 (0.75)]	[9.06 (0.85)]	[8.85 (0.74)]	**<0.001**
Log2-NLR	[1.04 (0.65)]	[1.21 (0.72)]	[1.02 (0.64)]	**<0.001**
Log2-PLR	[6.86 (0.55)]	[6.88 (0.64)]	[6.86 (0.54)]	0.6

^1^Mean ± SD for continuous; n (%) for categorical. ^2^Weighted *t*-tests for complex survey samples; chi-squared test with Rao & Scott’s second-order correction. Bold values indicate *p*-value < 0.05.

The baseline characteristics of Log2-SII, Log2-NLR, and Log2-PLR are summarized in Supplementary Tables S1–S3. We observed statistically significant differences in age, BMI, race, smoking status, diabetes, and cardiovascular disease across all three indices (all *p* < 0.05). Additionally, individuals with higher Log2-SII levels were more likely to be non-Hispanic white, had a higher propensity for obesity, and exhibited a greater likelihood of having diabetes and hypertension.

### Associations between SII, NLR, and PLR with COPD

The association between SII and other inflammatory markers with COPD is presented in Table 2. In the unadjusted model (crude model), Log2-SII and Log2-NLR exhibited a positive correlation with COPD, while Log2-PLR showed no significant relationship. When adjusting for age, gender, and race (Model 1), Log2-SII and Log2-NLR remained positively associated with COPD. Further adjustment for PIR, BMI, COT, education level, and smoking status (Model 2) revealed that only Log2-SII was positively associated with COPD. When all variables were adjusted in Model 3, both Log2-SII and Log2-PLR displayed a positive correlation with COPD, while Log2-NLR showed no significant relationship. Based on the above results, only the continuous variable SII consistently maintains a positive correlation with COPD. When SII and other inflammatory markers were converted from continuous variables to categorical variables, in all models, the highest SII group (Q4) consistently showed a positive association with COPD compared to the reference group (Q2).

**Table 2:** Association of SII, NLR, and PLR with COPD using weighted multivariate logistic analysis.

Index	Continuous or categories	Crude Model	Model 1	Model 2	Model3
		OR (95% CI)	OR (95% CI)	OR (95% CI)	OR (95% CI)
SII	Log2-SII	1.46 (1.26, 1.69)^***^	1.37 (1.18, 1.59)^***^	1.24 (1.05, 1.45)^*^	1.22 (1.04, 1.42)^*^
	Q1	1.14 (0.76, 1.70)	1.17 (0.80, 1.70)	1.26 (0.88, 1.80)	1.22 (0.84, 1.77)
	Q2	Reference	Reference	Reference	Reference
	Q3	1.39 (0.98, 1.97)	1.38 (0.97, 1.95)	1.33 (0.94, 1.88)	1.34 (0.94, 1.90)
	Q4	2.18 (1.66, 2.87)^***^	2.02 (1.52, 2.67)^***^	1.83 (1.36, 2.46)^***^	1.77 (1.32, 2.39)^***^
NLR	Log2-NLR	1.55 (1.34, 1.79)^***^	1.33 (1.14, 1.56)^***^	1.19(1.00,1.41)	1.12 (0.96, 1.32)
	Q1	0.69 (0.50, 0.95)^*^	0.70(0.50, 0.98)^*^	0.72 (0.53, 1.00)^*^	0.73 (0.53, 1.00)
	Q2	Reference	Reference	Reference	Reference
	Q3	0.81 (0.53, 1.24)	0.76 (0.48, 1.19)	0.75 (0.49, 1.14)	0.74 (0.48, 1.16)
	Q4	1.53 (1.12, 2.10)^**^	1.24 (0.89, 1.73)	1.04 (0.75, 1.44)	0.97 (0.69, 1.37)
PLR	Log2-PLR	1.06 (0.86, 1.29)	0.98 (0.81, 1.19)	1.21 (0.98, 1.50)	1.27 (1.04, 1.55)^*^
	Q1	1.29 (0.79, 2.10)	1.33 (0.81, 2.20)	1.09 (0.67, 1.79)	1.02 (0.63, 1.63)
	Q2	Reference	Reference	Reference	Reference
	Q3	1.01 (0.71, 1.42)	1.00 (0.72, 1.41)	1.10 (0.79, 1.53)	1.09 (0.79, 1.49)
	Q4	1.40 (0.99, 1.97)	1.27 (0.89, 1.80)	1.45 (1.04, 2.02)^*^	1.50 (1.06, 2.12)^*^

SII: Q1 (4.06–336.57), Q2 (336.71–465.38), Q3 (465.52–648.00), Q4 (648.21–6297.60); NLR: Q1 (0.037–1.545), Q2 (1.548–2.028), Q3 (2.033–2.700), Q4 (0.669–92.174); PLR: Q1 (92.22–93.87), Q2 (94.07–116.67), Q3 (116.79–145.38), Q4 (145.42–837.50). crude model, no covariates were adjusted. Model 1, age, sex, and race were adjusted. Model 2, age, sex, race, PIR, BMI, serum cotinine, education level, and smoking status were adjusted. Model 3, age, sex, race, PIR, BMI, serum cotinine, education level, smoking status, cardiovascular disease, hypertension, and diabetes were adjusted. 95% CI, 95% confidence interval; OR, odds ratio. *p < 0.05, **p < 0.01, ***p < 0.001; *p* < 0.05 was considered statistically significant.

**Figure 2:**
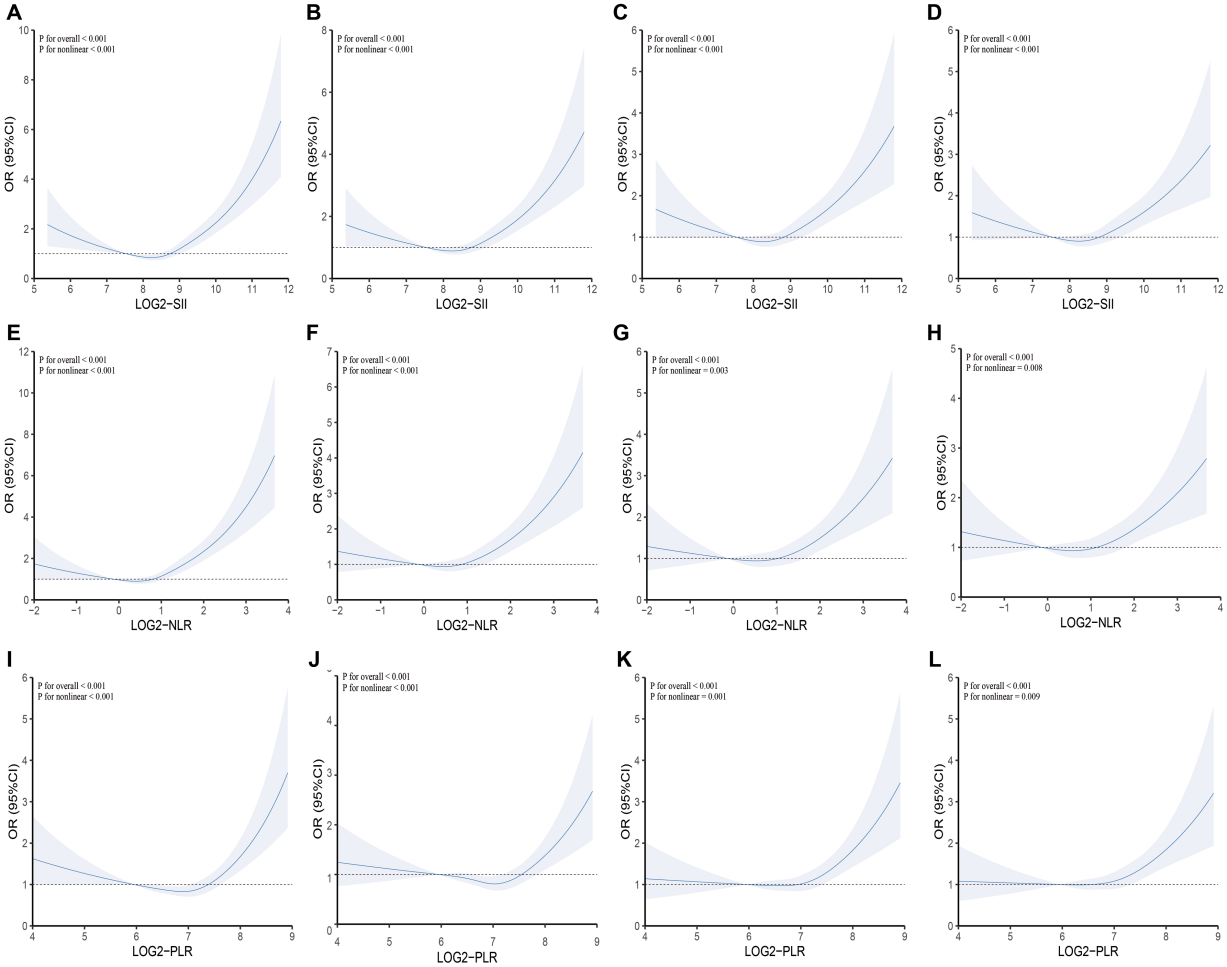
Testing for non-linearity of SII, NLR, and PLR in COPD analyses. **(A)** Crude model: SII and COPD; **(B)** Model 1: SII and COPD; **(C)** Model 2: SII and COPD; **(D)** Model 3: SII and COPD; **(E)** Crude model: NLR and COPD; **(F)** Model 1: NLR and COPD; **(G)** Model 2: NLR and COPD; **(H)** Model 3: NLR and COPD; **(I)** Crude model: PLR and COPD; **(J)** Model 1: PLR and COPD; **(K)** Model 2: PLR and COPD; **(L)** Model 3: PLR and COPD. Crude model: Unadjusted model; Model 1: Adjusted for age, sex, and race; Model 2: Adjusted for age, sex, race, PIR, BMI, serum cotinine, education level, and smoking status; Model 3: Adjusted for age, sex, race, PIR, BMI, serum cotinine, education level, smoking status, cardiovascular disease, hypertension, and diabetes. SII, NLR, and PLR were considered continuous variables (log2-SII, log2-NLR, log2-PLR).

**Figure 3:**
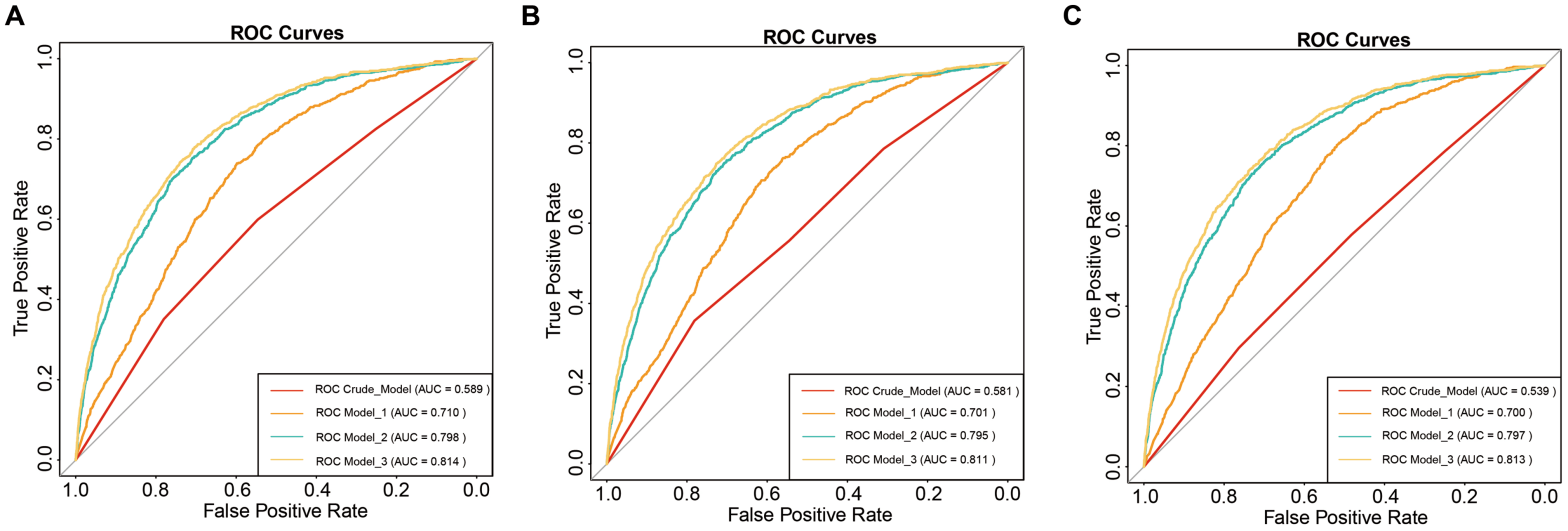
ROC curve analysis. **(A)** SII and COPD; **(B)** NLR and COPD; **(C)** PLR and COPD; Crude model: Unadjusted model; Model 1: Adjusted for age, sex, and race; Model 2: Adjusted for age, sex, race, PIR, BMI, serum cotinine, education level, and smoking status; Model 3: Adjusted for age, sex, race, PIR, BMI, serum cotinine, education level, smoking status, cardiovascular disease, hypertension, and diabetes.

Furthermore, we assessed the non-linear relationship between SII and other inflammatory markers with COPD. In particular, we observed that SII, NLR and PLR consistently exhibited a U-shaped relationship with COPD. Specific results are presented in Figure 2. Moreover, we conducted a comparative analysis using ROC curves for the inflammatory markers SII, NLR, and PLR. In the crude model, SII (AUC = 0.589) exhibited a higher predictive value for COPD prevalence compared to NLR (AUC = 0.581) and PLR (AUC = 0.539). For detailed information, please refer to Figure 3.

### Subgroup analyses

**Figure 4:**
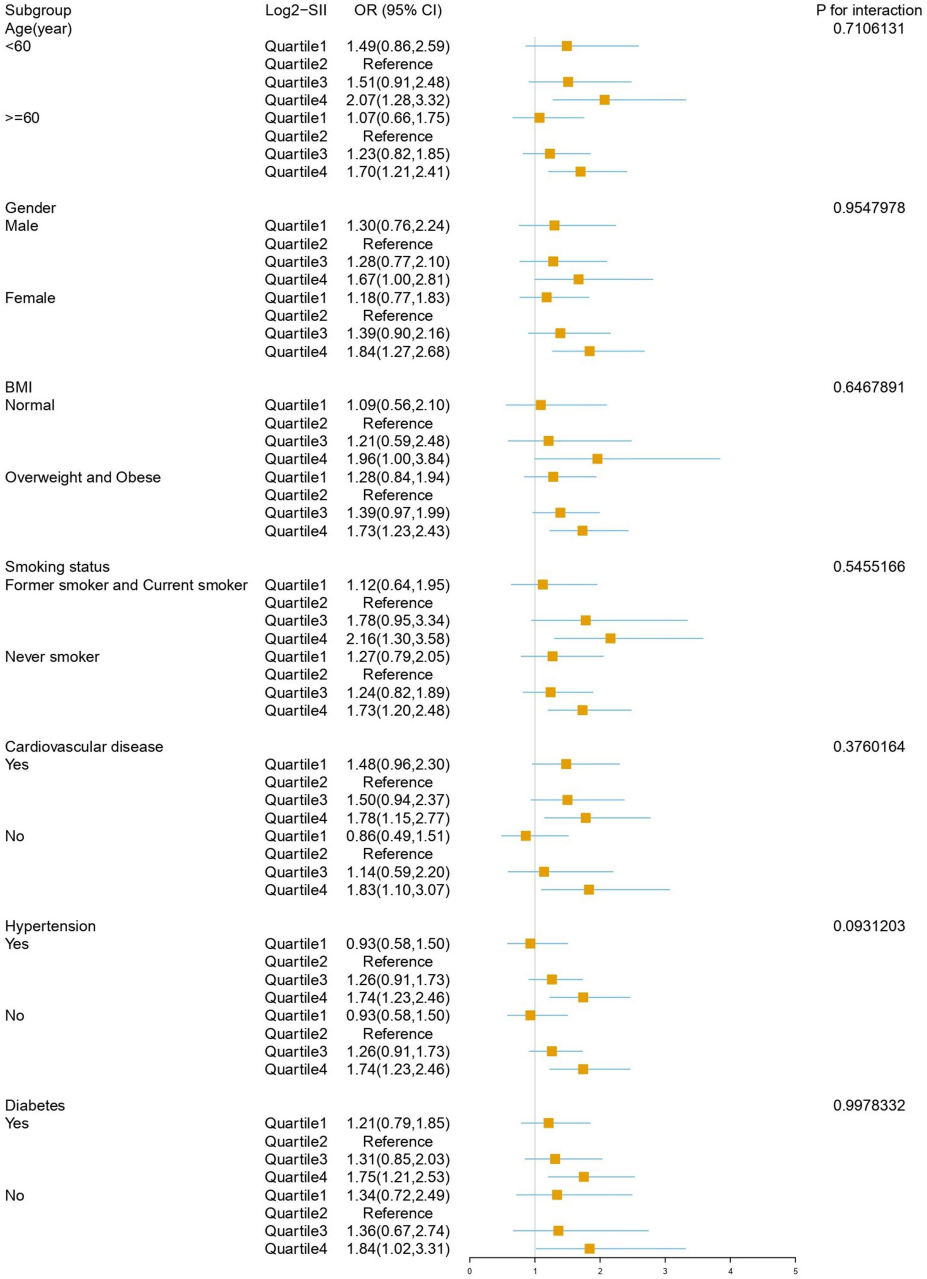
Subgroup analysis for the association between SII and COPD.

Subgroup analysis results after adjusting for all covariates are depicted in Figure 4. The impact of Log2-SII on COPD varies significantly across different subgroups of age, BMI, cardiovascular disease, hypertension, smoking status, and diabetes subgroups. Within the gender subgroup, a noteworthy association was observed only among female participants, where the highest quartile of SII had a 1.84-fold increased risk of COPD compared to the reference quartile (OR: 1.84, 95% CI: 1.27–2.68, *p* = 0.002), with no statistically significant association observed in males. Interaction testing indicated that age, gender, BMI, smoking status, diabetes, cardiovascular disease, and hypertension did not significantly influence this relationship (all interaction *p*-values >0.05).

## Discussion

This study found a positive association between SII and the risk of COPD. When SII was converted from a continuous variable to a categorical variable, individuals in the highest quartile of SII had a significantly higher risk of developing COPD compared to those in the reference quartile (OR: 1.84, 95% CI: 1.27–2.68) in the fully adjusted model. We also discovered a non-linear relationship between SII and COPD, which persisted after adjusting for covariates. Subgroup analysis revealed a more pronounced predictive effect of SII on COPD risk in females. Moreover, when compared to inflammatory markers such as NLR and PLR, SII demonstrated superior predictive capability for assessing COPD risk.

Peripheral blood ratio indices, such as NLR and PLR, have been shown to be associated with increased risk of COPD (13–15). This point was also confirmed in our study. Whether SII contributes to determining COPD risk is unclear. Previously, the SII has been applied in other respiratory diseases such as lung cancer and COVID-19. Wang’s et al. study showed that SII was an important prognostic factor for overall survival (OS) and progression-free survival (PFS) in patients with small cell lung cancer (SCLC) before treatment (16), which was also confirmed by a meta-analysis (17). Fois et al. explored the ability of various inflammatory indices, such as NLR, monocyte-to-lymphocyte ratio (MLR), PLR, and SII, to predict in-hospital mortality of COVID-19, and found that only SII remained significantly associated with survival after adjusting for confounding factors (18).

Our study confirmed a positive correlation between SII and COPD risk, yet the precise pathological and physiological mechanisms underlying SII and COPD remain unclear. From the perspective of SII, platelets participate in the inflammatory response, and activated platelets secrete a large number of inflammatory mediators into the nearby microenvironment, such as IL-1β, TNF-α. These mediators can further promote the migration of white blood cells to the site of inflammation, accelerating the occurrence of the two major mechanisms in COPD, namely the inflammatory response process and oxidative stress (19). Additionally, platelets also mediate the formation of pulmonary vascular microthrombi. Studies have suggested that COPD induces erythrocyte spherocytosis in patients, leading to the translocation (margination) of platelets to the vascular wall (20), promoting platelet adhesion, aggregation, and activation on the vascular wall (21). Platelet hyperaggregability may lead to arterial thrombosis, thereby further promoting the progression of chronic obstructive pulmonary disease. Neutrophils are the most abundant circulating white blood cells in the human blood, and are considered to be the first line of defense in innate immune defense. Their activation can release specific inflammatory mediators, leading to irreversible airway damage, such as neutrophil elastase, matrix metalloproteinase-9 (MMP-9), tissue protease G, Matrix metalloproteinases-48 (MMP-48), and myeloperoxidase (MPO), which can promote the pathological and physiological mechanisms of COPD (22). Neutrophil elastase can stimulate the production and secretion of mucin, leading to excessive mucus secretion and airway obstruction (23), while MPO can promote oxidative tissue damage and initiate cell homeostasis changes, and increase the response of lung epithelial cells to pro-inflammatory stimuli (24). Lymphocytes can cause alveolar destruction in COPD patients (25), among which CD8 cells produce pro-inflammatory cytokines, including IL-2, interferon-γ, and TNFα, which increase in COPD patients and recruit other inflammatory cells (26, 27). In addition, CD8 cells can release perforin, granzyme B, causing cell lysis and apoptosis of alveolar epithelial cells, thereby promoting the development of emphysema (28). However, from an epidemiological perspective, our evidence intuitively expresses the inherent connection between SII and COPD.

It is worth noting that our subgroup analysis revealed a stratified effect of gender on the association between SII and COPD. The underlying factors behind the gender difference in COPD are gradually being revealed, and the driving factors may include inflammatory pathways and harmful exposure. Women may exhibit more inflammation, such as higher levels of adipokines and IL-16 (29, 30), which have been found in COPD women (31). Analysis of 17, 139 patients from 22 COPD cohorts also indicated that women with COPD have a higher rate of deterioration (32).

This study has several strengths. Firstly, it is the first investigation of the association between SII levels and COPD risk in a representative US population. Additionally, it compares SII with inflammatory markers like NLR and PLR, enhancing its utility. Secondly, adjusting for confounding factors enhances the reliability and representativeness of the study; thirdly, robust and reliable conclusions were drawn in the subgroup analysis under the validation of interaction terms. However, this survey also has limitations. Due to the cross-sectional study design of NHANES, causal relationships cannot be inferred. Secondly, although we controlled for certain confounding factors, residual and unmeasured confounding cannot be ruled out. Thirdly, the diagnostic definition of COPD is based on self-report rather than more specific diagnostic methods, which may lead to recall bias. Fourthly, the correlation between SII and COPD is more significant in the US female population. Although this does not affect the overall effect, it is necessary to conduct further research to explore its potential mechanisms, which can contribute to precision medicine.

## Conclusion

As a novel inflammatory biomarker, SII can be employed to predict the risk of COPD among adults aged 40 and above in the United States, and it demonstrates superiority compared to NLR and PLR. Furthermore, a non-linear association exists between SII and the increased risk of COPD.

## Data availability statement

The original contributions presented in the study are included in the article/Supplementary material, further inquiries can be directed to the corresponding author.

## Ethics statement

The informed consent was provided by all NHANES survey participants before health examination. The study protocols were approved by the National Center for Health Statistics Research Ethics Review Committee.

## Author contributions

YX: Conceptualization, Data curation, Formal analysis, Project administration, Writing – original draft. ZY: Investigation, Software, Writing – original draft. KL: Investigation, Methodology, Writing – original draft. LL: Conceptualization, Funding acquisition, Project administration, Resources, Writing – review & editing.
