# Supplementary material for: The association between systemic immune-inflammation index and chronic obstructive pulmonary disease in adults aged 40 years and above in the United States: a cross-sectional study based on the NHANES 2013–2020
Source: Front Med (Lausanne). 2023 Nov 24;10:1270368. doi: 10.3389/fmed.2023.1270368 (PMC10704483; doi:10.3389/fmed.2023.1270368)
Supplement: Supplementary file 1 [file Table_1.docx]

TableS1 Baseline characteristics of study participants across Log2-SII groups

| **Characteristic** | **Overall**, N = 10364 (100%)^1^ | **Q1**, N = 2995 (25%)^1^ | **Q2**, N = 2540 (25%)^1^ | **Q3**, N = 2439 (25%)^1^ | **Q4**, N = 2390 (25%)^1^ | **P Value**^2^ |
| --- | --- | --- | --- | --- | --- | --- |
| **Age (years)** | 58.1 (11.5) | 58.0 (11.0) | 57.8 (11.4) | 57.6 (11.6) | 59.1 (12.0) | **0.011** |
| **Serum Cotinine (ng/mL)** | 56.5 (134.3) | 50.6 (127.2) | 54.9 (136.8) | 61.3 (145.9) | 59.3 (126.3) | 0.4 |
| **BMI** |  |  |  |  |  | **0.009** |
| *Normal (<25)* | 2,455 (24%) | 754 (26%) | 613 (24%) | 538 (22%) | 550 (24%) |  |
| *Overweight (≥25, <30)* | 3,510 (34%) | 1,068 (36%) | 876 (35%) | 834 (35%) | 732 (31%) |  |
| *Obese (≥30)* | 4,399 (42%) | 1,173 (38%) | 1,051 (41%) | 1,067 (44%) | 1,108 (45%) |  |
| **Sex** |  |  |  |  |  | 0.2 |
| *female* | 5,291 (52%) | 1,430 (50%) | 1,318 (52%) | 1,271 (53%) | 1,272 (55%) |  |
| *male* | 5,073 (48%) | 1,565 (50%) | 1,222 (48%) | 1,168 (47%) | 1,118 (45%) |  |
| **Race** |  |  |  |  |  | **<0.001** |
| *Non-Hispanic White* | 4,231 (71%) | 887 (62%) | 986 (70%) | 1,107 (75%) | 1,251 (78%) |  |
| *Non-Hispanic Black* | 2,306 (9.7%) | 995 (17%) | 512 (8.6%) | 444 (7.6%) | 355 (5.7%) |  |
| *Mexican American* | 1,287 (6.3%) | 347 (6.6%) | 344 (6.8%) | 311 (5.9%) | 285 (6.0%) |  |
| *Non-Hispanic Asian* | 1,123 (4.8%) | 373 (6.2%) | 342 (5.7%) | 242 (4.3%) | 166 (2.8%) |  |
| *Other Hispanic* | 1,080 (5.0%) | 294 (5.2%) | 282 (5.3%) | 254 (4.7%) | 250 (4.7%) |  |
| *Other/multiracial* | 337 (3.0%) | 99 (3.2%) | 74 (3.3%) | 81 (2.8%) | 83 (2.5%) |  |
| **PIR** |  |  |  |  |  | 0.6 |
| *High (>3.49)* | 3,487 (48%) | 1,002 (47%) | 867 (49%) | 842 (49%) | 776 (47%) |  |
| *Medium (>1.39, ≤3.49)* | 3,694 (33%) | 1,058 (33%) | 911 (33%) | 863 (33%) | 862 (32%) |  |
| *Low (≤1.39)* | 3,183 (19%) | 935 (20%) | 762 (18%) | 734 (18%) | 752 (20%) |  |
| **Education attainment** |  |  |  |  |  | 0.086 |
| *Less Than 9th Grade* | 992 (4.8%) | 307 (5.8%) | 278 (5.4%) | 225 (4.2%) | 182 (3.9%) |  |
| *9-11th Grade* | 1,206 (8.4%) | 349 (8.7%) | 279 (7.6%) | 283 (7.9%) | 295 (9.5%) |  |
| *High School Grad/GED* | 2,396 (23%) | 707 (22%) | 537 (21%) | 572 (25%) | 580 (24%) |  |
| *Some College or AA degree* | 3,153 (31%) | 850 (30%) | 778 (32%) | 757 (32%) | 768 (32%) |  |
| *College Graduate or above* | 2,617 (32%) | 782 (33%) | 668 (34%) | 602 (32%) | 565 (30%) |  |
| **Smoking status** |  |  |  |  |  | **0.015** |
| *Current smoker* | 1,821 (16%) | 481 (14%) | 407 (16%) | 458 (17%) | 475 (19%) |  |
| *Former smoker* | 2,992 (30%) | 834 (31%) | 723 (29%) | 699 (30%) | 736 (31%) |  |
| *Never smoker* | 5,551 (53%) | 1,680 (55%) | 1,410 (55%) | 1,282 (53%) | 1,179 (50%) |  |
| **Cardiovascular disease** | 1,644 (13%) | 446 (14%) | 344 (11%) | 396 (12%) | 458 (15%) | **0.040** |
| **Hypertension** | 7,149 (64%) | 2,040 (62%) | 1,695 (62%) | 1,664 (63%) | 1,750 (69%) | **0.003** |
| **Diabetes** | 2,217 (17%) | 569 (14%) | 558 (17%) | 480 (16%) | 610 (20%) | **0.002** |
| **COPD** |  |  |  |  |  | **<0.001** |
| *COPD* | 863.00 (7.26%) | 195.00 (5.91%) | 151.00 (5.24%) | 214.00 (7.14%) | 303.00 (10.76%) |  |
| *Non-COPD* | 9,501.00 (92.74%) | 2,800.00 (94.09%) | 2,389.00 (94.76%) | 2,225.00 (92.86%) | 2,087.00 (89.24%) |  |
| ^1^Mean ± SD for continuous; n (%) for categorical | | | | | | |
| ^2^Kruskal-Wallis rank-sum test for complex survey samples; chi-squared test with Rao & Scott's second-order correction | | | | | | |
| Bold values indicate P-value < 0.05. | | | | | | |

TableS2 Baseline characteristics of study participants across Log2-NLR groups

| **Characteristic** | **Overall**, N = 10364 (100%)^1^ | **Q1**, N = 3125 (25%)^1^ | **Q2**, N = 2416 (25%)^1^ | **Q3**, N = 2438 (25%)^1^ | **Q4**, N = 2385 (25%)^1^ | **P Value**^2^ |
| --- | --- | --- | --- | --- | --- | --- |
| **Age (years)** | 58.1 (11.5) | 56.9 (10.6) | 56.8 (11.3) | 57.8 (11.5) | 61.1 (12.1) | **<0.001** |
| **Serum Cotinine (ng/mL)** | 56.5 (134.3) | 51.9 (129.7) | 58.9 (140.7) | 54.1 (134.0) | 61.4 (132.4) | 0.7 |
| **BMI** |  |  |  |  |  | **0.025** |
| *Normal (<25)* | 2,455 (24%) | 748 (25%) | 580 (23%) | 548 (22%) | 579 (24%) |  |
| *Overweight (≥25, <30)* | 3,510 (34%) | 1,097 (37%) | 860 (35%) | 803 (34%) | 750 (31%) |  |
| *Obese (≥30)* | 4,399 (42%) | 1,280 (38%) | 976 (42%) | 1,087 (44%) | 1,056 (44%) |  |
| **Sex** |  |  |  |  |  | **<0.001** |
| *female* | 5,291 (52%) | 1,731 (58%) | 1,322 (55%) | 1,208 (52%) | 1,030 (46%) |  |
| *male* | 5,073 (48%) | 1,394 (42%) | 1,094 (45%) | 1,230 (48%) | 1,355 (54%) |  |
| **Race** |  |  |  |  |  | **<0.001** |
| *Non-Hispanic White* | 4,231 (71%) | 823 (57%) | 961 (72%) | 1,104 (75%) | 1,343 (80%) |  |
| *Non-Hispanic Black* | 2,306 (9.7%) | 1,109 (19%) | 458 (8.2%) | 407 (6.7%) | 332 (5.2%) |  |
| *Mexican American* | 1,287 (6.3%) | 363 (7.2%) | 332 (6.5%) | 328 (6.3%) | 264 (5.2%) |  |
| *Non-Hispanic Asian* | 1,123 (4.8%) | 401 (6.6%) | 319 (5.5%) | 262 (4.4%) | 141 (2.5%) |  |
| *Other Hispanic* | 1,080 (5.0%) | 317 (6.0%) | 278 (5.0%) | 257 (4.8%) | 228 (4.2%) |  |
| *Other/multiracial* | 337 (3.0%) | 112 (4.0%) | 68 (2.4%) | 80 (3.0%) | 77 (2.5%) |  |
| **PIR** |  |  |  |  |  | **0.020** |
| *High (>3.49)* | 3,487 (48%) | 1,032 (46%) | 865 (52%) | 838 (50%) | 752 (45%) |  |
| *Medium (>1.39, ≤3.49)* | 3,694 (33%) | 1,110 (33%) | 821 (31%) | 850 (32%) | 913 (35%) |  |
| *Low (≤1.39)* | 3,183 (19%) | 983 (21%) | 730 (18%) | 750 (18%) | 720 (20%) |  |
| **Education attainment** |  |  |  |  |  | **0.035** |
| *Less Than 9th Grade* | 992 (4.8%) | 319 (6.3%) | 235 (4.4%) | 243 (4.7%) | 195 (3.9%) |  |
| *9-11th Grade* | 1,206 (8.4%) | 351 (8.6%) | 277 (7.5%) | 282 (7.9%) | 296 (9.7%) |  |
| *High School Grad/GED* | 2,396 (23%) | 704 (21%) | 559 (23%) | 542 (22%) | 591 (25%) |  |
| *Some College or AA degree* | 3,153 (31%) | 932 (31%) | 723 (31%) | 739 (31%) | 759 (32%) |  |
| *College Graduate or above* | 2,617 (32%) | 819 (33%) | 622 (34%) | 632 (34%) | 544 (29%) |  |
| **Smoking status** |  |  |  |  |  | **<0.001** |
| *Current smoker* | 1,821 (16%) | 528 (15%) | 410 (16%) | 439 (16%) | 444 (19%) |  |
| *Former smoker* | 2,992 (30%) | 771 (27%) | 669 (30%) | 729 (30%) | 823 (34%) |  |
| *Never smoker* | 5,551 (53%) | 1,826 (58%) | 1,337 (54%) | 1,270 (54%) | 1,118 (47%) |  |
| **Cardiovascular disease** | 1,644 (13%) | 376 (11%) | 326 (11%) | 374 (12%) | 568 (19%) | **<0.001** |
| **Hypertension** | 7,149 (64%) | 2,084 (60%) | 1,621 (62%) | 1,683 (64%) | 1,761 (69%) | **<0.001** |
| **Diabetes** | 2,217 (17%) | 581 (14%) | 461 (14%) | 525 (18%) | 650 (21%) | **<0.001** |
| **COPD** |  |  |  |  |  | **<0.001** |
| *COPD* | 863.00 (7.26%) | 185.00 (5.11%) | 172.00 (7.27%) | 198.00 (5.97%) | 308.00 (10.73%) |  |
| *Non-COPD* | 9,501.00 (92.74%) | 2,940.00 (94.89%) | 2,244.00 (92.73%) | 2,240.00 (94.03%) | 2,077.00 (89.27%) |  |
| ^1^Mean ± SD for continuous; n (%) for categorical | | | | | | |
| ^2^Kruskal-Wallis rank-sum test for complex survey samples; chi-squared test with Rao & Scott's second-order correction | | | | | | |
| Bold values indicate P-value < 0.05. | | | | | | |

TableS3 Baseline characteristics of study participants across Log2-PLR groups

| **Characteristic** | **Overall**, N = 10364 (100%)^1^ | **Q1**, N = 2909 (25%)^1^ | **Q2**, N = 2549 (25%)^1^ | **Q3**, N = 2397 (25%)^1^ | **Q4**, N = 2509 (25%)^1^ | **P Value**^2^ |
| --- | --- | --- | --- | --- | --- | --- |
| **Age (years)** | 58.1 (11.5) | 57.7 (11.3) | 57.7 (11.3) | 57.5 (11.5) | 59.6 (11.9) | **<0.001** |
| **Serum Cotinine (ng/mL)** | 56.5 (134.3) | 79.1 (152.5) | 55.0 (129.7) | 48.7 (123.5) | 43.2 (126.7) | **<0.001** |
| **BMI** |  |  |  |  |  | **<0.001** |
| *Normal (<25)* | 2,455 (24%) | 578 (20%) | 586 (21%) | 559 (24%) | 732 (30%) |  |
| *Overweight (≥25, <30)* | 3,510 (34%) | 978 (34%) | 866 (34%) | 821 (35%) | 845 (35%) |  |
| *Obese (≥30)* | 4,399 (42%) | 1,353 (47%) | 1,097 (45%) | 1,017 (41%) | 932 (35%) |  |
| **Sex** |  |  |  |  |  | **<0.001** |
| *female* | 5,291 (52%) | 1,354 (48%) | 1,273 (51%) | 1,280 (55%) | 1,384 (57%) |  |
| *male* | 5,073 (48%) | 1,555 (52%) | 1,276 (49%) | 1,117 (45%) | 1,125 (43%) |  |
| **Race** |  |  |  |  |  | **<0.001** |
| *Non-Hispanic White* | 4,231 (71%) | 1,019 (64%) | 1,005 (71%) | 1,057 (75%) | 1,150 (75%) |  |
| *Non-Hispanic Black* | 2,306 (9.7%) | 723 (12%) | 532 (8.8%) | 460 (7.5%) | 591 (10%) |  |
| *Mexican American* | 1,287 (6.3%) | 417 (8.1%) | 349 (6.6%) | 281 (5.8%) | 240 (4.7%) |  |
| *Non-Hispanic Asian* | 1,123 (4.8%) | 319 (5.4%) | 304 (4.9%) | 285 (4.9%) | 215 (3.8%) |  |
| *Other Hispanic* | 1,080 (5.0%) | 319 (5.7%) | 277 (5.1%) | 240 (4.8%) | 244 (4.4%) |  |
| *Other/multiracial* | 337 (3.0%) | 112 (4.1%) | 82 (3.2%) | 74 (2.4%) | 69 (2.2%) |  |
| **PIR** |  |  |  |  |  | **<0.001** |
| *High (>3.49)* | 3,487 (48%) | 839 (39%) | 850 (49%) | 883 (52%) | 915 (52%) |  |
| *Medium (>1.39, ≤3.49)* | 3,694 (33%) | 1,068 (38%) | 944 (33%) | 812 (31%) | 870 (30%) |  |
| *Low (≤1.39)* | 3,183 (19%) | 1,002 (23%) | 755 (18%) | 702 (17%) | 724 (18%) |  |
| **Education attainment** |  |  |  |  |  | **<0.001** |
| *Less Than 9th Grade* | 992 (4.8%) | 360 (7.0%) | 248 (4.6%) | 196 (4.2%) | 188 (3.6%) |  |
| *9-11th Grade* | 1,206 (8.4%) | 364 (9.8%) | 310 (8.5%) | 254 (7.5%) | 278 (7.8%) |  |
| *High School Grad/GED* | 2,396 (23%) | 700 (25%) | 576 (23%) | 562 (23%) | 558 (21%) |  |
| *Some College or AA degree* | 3,153 (31%) | 892 (33%) | 747 (30%) | 744 (31%) | 770 (31%) |  |
| *College Graduate or above* | 2,617 (32%) | 593 (25%) | 668 (33%) | 641 (35%) | 715 (37%) |  |
| **Smoking status** |  |  |  |  |  | **<0.001** |
| *Current smoker* | 1,821 (16%) | 658 (23%) | 462 (17%) | 349 (14%) | 352 (12%) |  |
| *Former smoker* | 2,992 (30%) | 809 (30%) | 739 (31%) | 682 (28%) | 762 (32%) |  |
| *Never smoker* | 5,551 (53%) | 1,442 (47%) | 1,348 (53%) | 1,366 (57%) | 1,395 (56%) |  |
| **Cardiovascular disease** | 1,644 (13%) | 499 (16%) | 374 (12%) | 378 (12%) | 393 (12%) | **0.006** |
| **Hypertension** | 7,149 (64%) | 2,058 (66%) | 1,763 (65%) | 1,601 (61%) | 1,727 (63%) | 0.053 |
| **Diabetes** | 2,217 (17%) | 706 (21%) | 563 (17%) | 485 (16%) | 463 (13%) | **<0.001** |
| **COPD** |  |  |  |  |  | 0.15 |
| *COPD* | 863.00 (7.26%) | 243.00 (7.92%) | 184.00 (6.27%) | 180.00 (6.31%) | 256.00 (8.54%) |  |
| *Non-COPD* | 9,501.00 (92.74%) | 2,666.00 (92.08%) | 2,365.00 (93.73%) | 2,217.00 (93.69%) | 2,253.00 (91.46%) |  |
| ^1^Mean ± SD for continuous; n (%) for categorical | | | | | | |
| ^2^Kruskal-Wallis rank-sum test for complex survey samples; chi-squared test with Rao & Scott's second-order correction | | | | | | |
| Bold values indicate P-value < 0.05. | | | | | | |
